# The shift from high to low turnover bone disease after parathyroidectomy is associated with the progression of vascular calcification in hemodialysis patients: A 12-month follow-up study

**DOI:** 10.1371/journal.pone.0174811

**Published:** 2017-04-06

**Authors:** Fabiana Rodrigues Hernandes, Maria Eugênia Fernandes Canziani, Fellype Carvalho Barreto, Rodrigo Oliveira Santos, Valéria de Melo Moreira, Carlos Eduardo Rochitte, Aluizio Barbosa Carvalho

**Affiliations:** 1 Nephrology Division, Federal University of Sao Paulo, Sao Paulo, Brazil; 2 Nephrology Division, Federal University of Parana, Parana, Brazil; 3 Head and Neck Surgery Division, Federal University of Sao Paulo, Sao Paulo, Brazil; 4 Cardiology Division, University of Sao Paulo, Sao Paulo, Brazil; Emory University Department of Medicine, UNITED STATES

## Abstract

Parathyroidectomy (PTX) may cause low levels of PTH, leading to an excessive reduction of bone turnover, which is associated with poor outcomes in dialysis patients, including vascular calcification (VC). We aimed to prospectively investigate the impact of PTX on bone remodeling and its potential consequence on the progression of VC in hemodialysis patients. In this prospective study, 19 hemodialysis patients with severe secondary hyperparathyroidism (sHPT) were evaluated. All patients underwent laboratorial tests and coronary tomography at baseline and, 6 and 12 months after PTX; bone biopsy was performed at baseline and 12-month. At baseline, all patients had increased PTH levels up to 2500 pg/mL and high turnover bone disease in their bone biopsies. Fourteen (74%) patients had VC. During the follow-up, there was a significant decrease of PTH at 6 and 12-month. At 12-month, 90% of the patients evolved to low turnover bone disease. During the period of the hungry bone syndrome (first 6 months), no change of coronary calcium score was observed. However, calcium score increased significantly thereafter (12^th^ month). There was an association between VC progression and the severity of low turnover bone disease. In conclusion, the shift from high to low turnover bone disease after PTX occurs in parallel to VC progression, contributing to the understanding of the complex pathophysiology involving mineral metabolism and cardiovascular disease in hemodialysis patients.

## Introduction

Secondary hyperparathyroidism (sHPT) is a frequent and severe co-morbidity among chronic kidney disease (CKD) patients, which is associated with cardiovascular disease, the main cause of the high rate of mortality in this population [1–6]. Although the current pharmacological treatment has changed the course of sHPT, severe forms of the disease still require invasive procedures, such as parathyroidectomy (PTX) [7]. In the setting of severe sHPT, PTX has been considered to provide clinical short term benefits. However, the post-PTX resultant low levels of PTH may lead to an excessive reduction of bone turnover, which is associated with poor outcomes in dialysis patients. In fact, it has been demonstrated, in cross-sectional studies, that low turnover bone disease is related to vascular calcification (VC) in both non-dialyzed and dialyzed CKD patients [8–9]. It is well known that VC is a frequent and severe comorbidity in CKD patients [10–14]. Of note, not only the presence of VC, but also its progression has been associated with high mortality rate [15–16]. Thus, we aimed to prospectively investigate the impact of PTX on bone remodeling and its potential consequence on the progression of VC in hemodialysis patients.

## Patients and methods patients

Hemodialysis patients referred to PTX due to severe sHPT were approached to participate in this study. Exclusion criteria were age below 18 or above 65 years, presence of positive deferroxamine test, serious gastrointestinal disease, ethanol or drug abuse, active malignancy, human immunodeficiency virus infection, chronic inflammatory disease, previous myocardial revascularization, uncontrolled diabetes mellitus or hypertension, body weight greater than 100 kg, pregnancy or breast-feeding, and use of corticosteroids, antiarrhythmic or seizure drugs. Nineteen out of 25 screened patients completed a 12-month follow-up. The reasons of drop-out were: death (2 patients), persistent sHPT (2 patients), withdraw (1 patient) and lung tuberculosis (1 patient). These patients were not different regarding baseline demographic, laboratory, coronary tomography or bone biopsy characteristics from those who completed the study.

The study was reviewed and approved by the Ethics Advisory Committee of the Federal University of São Paulo (approval number: 0918/06). All patients gave written informed consent.

### Study protocol

This was a prospective study of 12 months. At the time of PTX (baseline), all patients underwent an assessment of their clinical history, physical examination, laboratory tests, coronary tomography and bone biopsy. Laboratory tests and coronary tomography were repeated at 6 and 12-month, and the bone biopsy at 12-month. During the follow-up, cumulative doses of calcitriol and elemental calcium from calcium salts were recorded.

According to local protocol, patients were referred to PTX due to unsuccessful pharmacological therapy or the presence of severe bone pain, fractures and calciphylaxis. Total PTX followed by parasternal autotransplantation was performed as described elsewhere [17].

### Multislice coronary tomography

In order to evaluate VC, patients underwent coronary calcium quantification by multislice computed tomography scanner (Somatrom Volum Zoom Siemens AG®, Erlhagen, Germany) using a gantry rotation of 0.4s, collimation of 2.5mm (slice thickness), and reconstruction time of 6 frames per second. All obtained scans were analyzed using an Indigo 2^®^ workstation (SGI, Mountain View, CA, USA) and a calcium threshold of ≥ 130 Housfield Units was used. The images were scored by a single radiologist blinded to all clinical and biochemical data of the patients. Total score was the sum of each coronary score, expressed in modified Agatston Units (AU) [18]. Presence of VC was defined as coronary calcium score >10 AU. The absolute progression of VC, named as delta-calcium score, was calculated as the difference between 12-month and baseline calcium score.

### Bone biopsy

Baseline and 12-month bone specimens were obtained from the iliac crest. The second biopsy was performed in the contralateral site to avoid interference of repair processes from the previous biopsy. The procedure was conducted using a trephine with a 7-mm inner diameter adapted to an electrical drill (Gauthier Medical, Rochester, MN, USA). All patients were prelabeled with oral tetracycline (20 mg/kg/d for 3 days) administered over 2 periods, 10 days apart. Undecalcified bone fragments were submitted to specific histological technique, described elsewhere [19]. Bone histomorphometric analysis was conducted using the semiautomatic method contained in the Osteomeasure software (Osteometrics Inc, Atlanta, GA, USA). Histomorphometric parameters were those suggested by the American Society of Bone and Mineral Research histomorphometry nomenclature committee [20]. Reference values (RV) used for static parameters were obtained from local controls [21], whereas dynamic parameters followed those described elsewhere [22].

The following parameters were analyzed: trabecular bone volume/total bone volume (BV/TV; RV = 24 ± 6.1% for men and 21.8 ± 7.2% for women), osteoid volume/trabecular bone volume (OV/BV; RV = 2.9 ± 2.7% for men and 1.55 ± 1.9% for women), trabecular thickness (TbTh; RV = 127.9 ± 29.7 μm for men and 126 ± 28.8 μm for women), trabecular separation (TbSp; RV = 420.6 ± 124.1 μm for men and 498.3 ± 195.9 μm for women), trabecular number (TbN; RV = 1.89 ± 0.42 N/mm for men and 1.76 ± 0.52 N/mm for women), osteoblast surface/bone surface (Ob.S/BS; RV = 1.2 ± 1.4% for men and 1.2 ± 3.2% for women), osteoclast surface/bone surface (Oc.S/BS; RV = 0.03 ± 0.11% for men and 0.03 ± 0.06% for women), eroded surface/bone surface (ES/BS; RV = 2.3 ± 2.4% for men and 1.75 ± 1.21% for women), marrow fibrosis volume/total bone volume (Fb.V/TV; RV = 0%), bone formation rate/bone surface (BFR/BS; RV = 0.13± 0.07 μm^3^/μm^2^/d for men and 0.07 ± 0.03 μm^3^/μm^2^/d for women), mineralization lag time (Mlt; RV = 23.7 ± 2.7 days for men and 21.3 ± 2.3 days for women). Patients were classified according to BFR/BS into high turnover bone disease (HTBD = BFR/BS values greater than 1 standard deviation (SD) above the normal range) or low turnover bone disease (LTBD = BFR/BS values greater than 1 SD below the normal range) [23]. Patients whose trabecular bone surface did not uptake tetracycline were classified as having very low turnover bone disease (VLTBD).

### Laboratory analyses

Blood samples were drawn after an overnight fast of at least 12 hours for determining the following laboratory tests: ionized calcium, phosphorus, total alkaline phosphatase (35–104 U/L for females and 40–129 U/L for males), intact PTH (Immulite; DPC-Biermann, Bad Nauheim, Germany, 10–65 pg/ml), intact fibroblast growth factor 23 (FGF23; Elisa assay, Kainos, Japan) at baseline and 12-month.

### Statistical analyses

Mean and standard deviation, median and interquartile range or frequencies were calculated for all variables. Comparisons of continuous variables were done by Student´s or Wilcoxon tests according to the variable distribution. In order to evaluate the changes of calcium score in the patients with VC at baseline, a repeated measure design was used considering three periods (baseline, 6 and 12-month) as the main effect. For those with normal distribution, test for time was conducted using ANOVA for repeated measures via MIXED procedure, followed by adjusted Tukey test for multiple comparisons. When the variable did not have normal distribution, the same design was used fitting a generalized linear model via GENMOD procedure with gamma distribution and link reciprocal. Multiple comparisons in this case were made using DIFF option from the GENMOD procedure [24]. Spearman´s correlation test was applied to evaluate the association between delta-calcium score and cumulative doses of elemental calcium and calcitriol. All procedures were made using SAS for Windows (version 9.2; SAS, Cary, NC, USA), and *p* value < 0.05 was considered statistically significant.

## Results

Demographic, laboratory, bone biopsy and coronary tomography parameters at baseline, 6 and 12-month from the 19 patients who completed the study are shown in Table 1. Patients were relatively young, white women, long time on dialysis and 63% with BMI < 23 kg/m^2^. Most of the patients had hypertension (68%) and none had diabetes as comorbid condition. Six patients were not submitted to autotransplantation after PTX due to the absence of suitable parathyroid tissue.

**Table 1:** Demographic, coronary tomography, laboratory and bone histomorphometry data of 19 patients.

	*Baseline*	*6-month*	*12-month*
**Demographic characteristics**			
Age (y)	45 ± 11		
Women n (%)	12 (63)		
White n (%)	13 (68)		
Time on hemodialysis (mo)	105 ± 46		
PTX+autotransplantation (%)	68		
Body mass index (Kg/m^2^)	23.1 ± 3.8		23.1 ± 3.6
**Laboratory**			
Ionized calcium (mmol/L)	1.33 ± 0.10	1.21 ± 0.11^a^	1.19 ± 0.11 ^a^
Phosphorus (mg/dl)	6.8 ± 2.1	4.9 ± 2.1^a^	5.3 ± 1.6
Alkaline phosphatase (U/L)	565 (377–984)	107 (82–146)^a^	106 (73–126) ^a^
Intact PTH (pg/ml)	2152 (1638–2500)	47 (10–71)^a^	58 (12–117) ^a^
FGF23 (pg/ml)	8583 (2879–59283)		6701(134–18396)
**Bone Histomorphometry**			
BV/TV (%)	11.3 (7.7–16)		14.9 (8.5–20)
TbTh (μm)	99.3 (85.7–127.3)		137.8 (114.5–156) ^a^
TbSp (%)	750 (553–1155)		796 (514–1365)
TbN (n)	1.1 (0.7–1.5)		0.6 (0.67–1.45)
OV/BV (%)	8.9 (5.7–14)		7.2 (2–8.8) ^a^
Ob.S/BS (%)	7.9 (6.8–18.6)		1.04 (0.5–1.7) ^a^
ES/BS (%)	16.1 (13.2–20.4)		1.7 (0.9–3.5) ^a^
Oc.S/BS (%)	5.9 (4.4–6.8)		0.3 (0.1–0.6) ^a^
Fb.V/TV (%)	5.1 (2–7.8)		0.01 (0–0.1) ^a^
BFR/BS (μm^3^/ μm^2^/d)	0.16 (0.09–0.22)		0.006 (0.006–0.009) ^a^
Mlt (d)	32 (21–52)		809 (466–809) ^a^
**Coronary Tomography**			
Calcium score (AU)	76 (2.2–996)	103 (2.2–1082)	122 (2.4–1160) ^a^^,^^b^

Mean ±standard deviation, median and interquartile range or frequencies.

a: vs.baseline

b: vs. 6-month; p<0.05.

Abbreviations: PTX, parathyroidectomy; PTH, parathormone; FGF23, fibroblast growth factor 23; BV/TV, trabecular bone volume / total volume; TbTh, trabecular thickness; TbSp, trabecular separation; TbN, trabecular number; OV/BV, osteoid volume / trabecular bone volume; Ob.S/BS, osteoblastic surface / bone surface; ES/BS, eroded surface / bone surface; Oc.S/BS, osteoclastic surface / bone surface; Fb.V/TV, marrow fibrosis volume / total volume; BFR/BS, bone formation rate / bone surface; Mlt, mineralization lag time

At baseline, laboratory data are in accordance with severe sHPT, with increased PTH levels up to 2500 pg/mL. At 6 and 12-month, patients had significant lower levels of serum ionized calcium, alkaline phosphatase and PTH compared to baseline. Phosphorus levels had a significant decrease only at 6-month while FGF23 remained stable.

**Fig 1:**
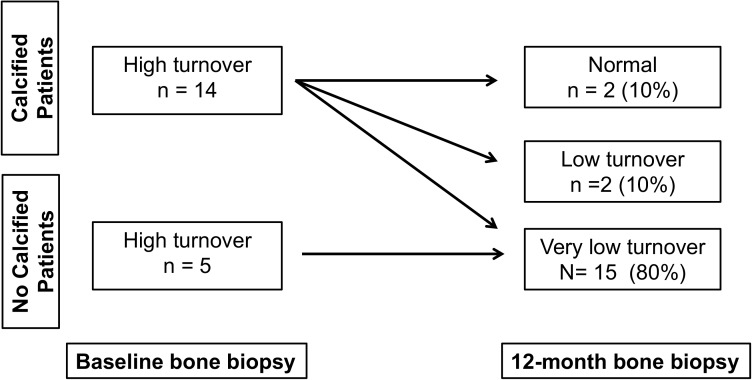
Evolution of bone turnover in the calcified and no calcified patients following PTX.

Regarding renal osteodystrophy (ROD), at the baseline, all patients had HTBD, as shown by the increased values of osteoid volume, osteoblastic, eroded and osteoclastic surfaces, fibrosis volume and bone formation rate. Of note, aluminum deposits on bone surface were not detected. At 12-month, there was a significant decrease of all those histomorphometric parameters, while an increase of trabecular thickness and mineralization lag time, and a trend to an increase of bone volume (p = 0.09) were observed. According to the ROD diagnose, 10% of the patients evolved to normal bone turnover, 10% to LTBD and 80% to VLTBD (Fig 1).

**Fig 2:**
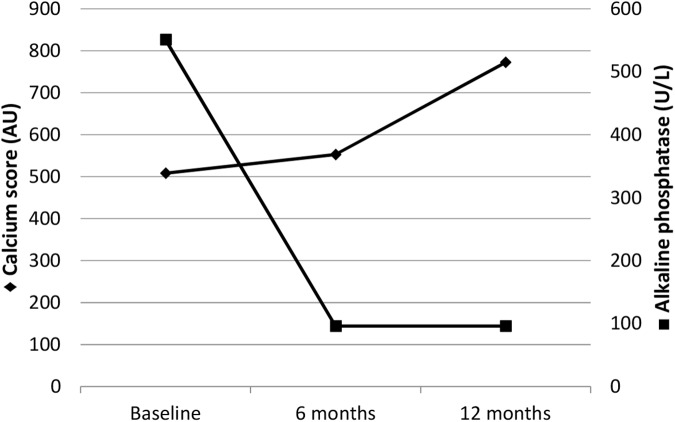
Calcium score and alkaline phosphatase in hemodialysis patients with VC during 12 months after parathyroidectomy (n = 14).

**Fig 3:**
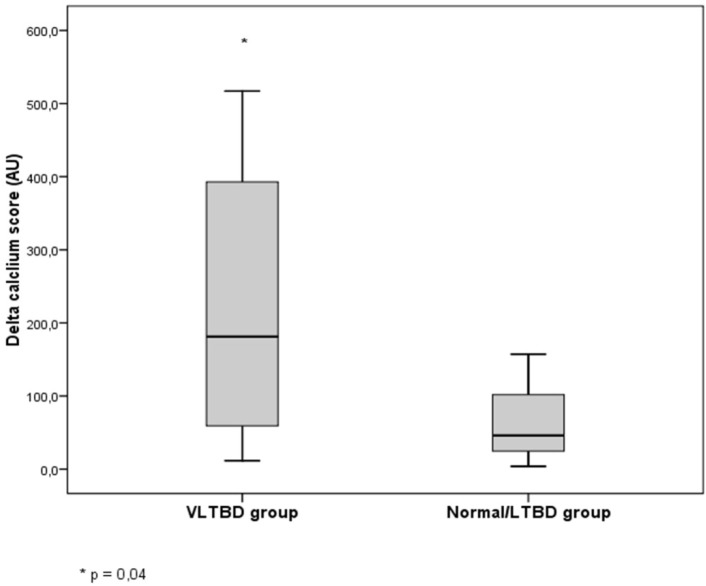
Comparison of delta calcium score between very low (VLTBD) and normal/low (LTBD) turnover bone disease groups.

At baseline, 5 out of 19 patients (26%) had no VC, remaining unchanged until the end of the study. Regarding clinical, demographic and laboratorial parameters, this group was similar to the 14 calcified patients. Of note, progression was observed only in patients who had VC at baseline (n = 14). In this calcified group there was no significant difference between calcium score at baseline and 6-month. However, a significant increase of calcium score was observed at 12-month when compared to baseline and 6-month. Fig 2 shows the changes of calcium score and alkaline phosphatase of these patients during the study. Total alkaline phosphatase decreased during the first 6 months [from 551 (355–788) at baseline to 96 (79–143) U/L at 6-month (p = 0.01)], while calcium score increased during the last 6 months after PTX [from 552 (65–1308) at 6-month to 772 (90–1397) AU at 12-month; p = 0.02]. The doses of elemental calcium and calcitriol in the first 6 months were significantly higher than those ones in the last 6 months [241 (188–244) *vs*. 135 (73–252) g; p<0.01 and 257 (192–381) *vs*. 34.3 (5.2–101) mcg, respectively; p = 0.001]. Of note, no correlation was found between delta-calcium score and the cumulative doses of both drugs. In the patients with VC progression, 14% evolved to normal bone turnover, 14% to LTBD and 72% to VLTBD. The delta-calcium score of the VLTBD compared to normal plus LTBD groups was significantly higher [197 AU (53–396) *vs*. 46 AU (14–128), respectively; p = 0.04] (Fig 3).

## Discussion

In the current study, we could observe that the shift from high to low turnover bone disease after PTX was related to the progression of VC in hemodialysis patients.

Parathyroidectomy has been still proposed as a useful treatment for severe sHPT, even after the introduction of drugs such as cinacalcet and selective activators of vitamin D receptor. Parathyroid hormone levels have been shown to be low shortly after PTX [25,26]. In the present study, the PTH values at 12-month were low and similar to those reported by Fotheringham et al [26]. These authors described a cohort of 252 CKD patients whose median of PTH levels were 139 (interquartile range 46–420) pg/mL after 5 years following PTX. Although clinical short term benefits of PTX have been unquestionable, the drastic decrease of PTH levels, even in those patients underwent autotransplantation, may lead to an extreme reduction of bone turnover [8]. It is well known that PTH levels lower than 150 pg/mL is associated with LTBD in patients on dialysis [27–29]. In fact, in the present study > 90% reduction of PTH levels occurred along with all but two patients who developed LTBD in the period of one year after PTX. Moreover, the shift from high to low bone turnover status was well documented. After one year of PTX, the observed decrease of bone formation and resorption parameters, e.g., paucity of cellularity, lacking of marrow fibrosis and the striking reduction of bone formation rate corroborate with the diagnosis of LTBD, as described elsewhere [8,30]. This exchange in bone turnover patterns after PTX comprises a period of increasing bone formation in parallel with cessation of bone resorption, named “hungry bone syndrome”, characterized by the decrease of alkaline phosphatase, serum calcium and phosphorus levels, associated to requirements of high doses of calcium salts and calcitriol [31]. In this cohort, the laboratorial characteristics of this phenomenon could be observed in the first 6 months after PTX, accompanied by a significant decrease of alkaline phosphatase levels, as depicted in Fig 2.

In the first 6-month period, VC progression did not evolve even under the use of high doses of calcium salts and calcitriol. This finding could be partially explained by the improvement of bone tissue buffering capability, which favors the accretion of calcium and phosphorus into the bone matrix. Interestingly, VC progression could be observed during the second 6 months after PTX, in which “hungry bone syndrome” has finished, as suggested by the normalization of alkaline phosphatase. During this period, lesser but not low doses of calcium and calcitriol are still necessary in order to maintain the serum calcium within the normal range. This may have influenced the VC progression. However, this hypothesis could not be established in this study, as no correlation was observed between the change of calcium score and the cumulative doses of calcium and calcitriol.

An unquestionable contribution of this study to better understand the events formerly discussed was the availability of two consecutive bone biopsies. Although the association of LTBD with VC has been suggested by London et al in a cross-sectional study [8], as far as we are concerned, the present study is the first one to demonstrate prospectively this association. One could speculate about the relationship between the exchange pattern of bone biopsy and VC. In fact, the shift from HTBD to LTBD in parallel with VC progression could advocate this hypothesis. Furthermore, once VC progression occurs only in the last 6-month period, e.g., after the cessation of the “hungry bone syndrome”, we could consider that LTBD has emerged at this moment. Unfortunately, the lack of bone biopsy at 6-month does not allow us to confirm that hypothesis. Furthermore, we could demonstrate herein the association between the severity of low turnover bone disease and the progression of VC. The finding of a subgroup of patients with VLTBD and higher delta calcium score at 12-month, suggests that the lower bone turnover the higher VC progression (Fig 3). It is important to mention that the absence of tetracycline labels in the trabecular bone of patients with VLTBD could not be ascribed to the failure of tetracycline intake, as we could find tetracycline double labels in the cortical bone of these patients (data not shown).

The limitations of the current study rely on the relatively few patients enrolled into the investigation which was due to the strict inclusion criteria (patients with severe sHPT referred to PTX), and on the lack of a control group, once the maintenance of such a group of patients without treatment would conflict to ethical issues.

In conclusion, in this study, the particular pattern of VC progression and the parallel association with the decreasing bone turnover, observed after PTX, could add a contribution for the understanding of the complex pathophysiology involving mineral metabolism and cardiovascular disease in hemodialysis patients.
